# Non-thermal Plasma Exposure Rapidly Attenuates Bacterial AHL-Dependent Quorum Sensing and Virulence

**DOI:** 10.1038/srep26320

**Published:** 2016-05-31

**Authors:** Padrig B. Flynn, Alessandro Busetti, Ewa Wielogorska, Olivier P. Chevallier, Christopher T. Elliott, Garry Laverty, Sean P. Gorman, William G. Graham, Brendan F. Gilmore

**Affiliations:** 1 Biofilm Research Group, School of Pharmacy, Queen’s University Belfast, BT9 7BL, UK; 2 Centre for Plasma Physics, School of Maths and Physics, Queen’s University Belfast, BT7 1NN, UK; 3 Advanced Asset Centre, Institute for Global Food Security, School of Biological Sciences, Queen’s University Belfast, 18-30 Malone Road, Belfast, BT9 5BN, UK.

## Abstract

The antimicrobial activity of atmospheric pressure non-thermal plasma has been exhaustively characterised, however elucidation of the interactions between biomolecules produced and utilised by bacteria and short plasma exposures are required for optimisation and clinical translation of cold plasma technology. This study characterizes the effects of non-thermal plasma exposure on acyl homoserine lactone (AHL)-dependent quorum sensing (QS). Plasma exposure of AHLs reduced the ability of such molecules to elicit a QS response in bacterial reporter strains in a dose-dependent manner. Short exposures (30–60 s) produce of a series of secondary compounds capable of eliciting a QS response, followed by the complete loss of AHL-dependent signalling following longer exposures. UPLC-MS analysis confirmed the time-dependent degradation of AHL molecules and their conversion into a series of by-products. FT-IR analysis of plasma-exposed AHLs highlighted the appearance of an OH group. *In vivo* assessment of the exposure of AHLs to plasma was examined using a standard *in vivo* model. Lettuce leaves injected with the *rhlI/lasI* mutant PAO-MW1 alongside plasma treated N-butyryl-homoserine lactone and n-(3-oxo-dodecanoyl)-homoserine lactone, exhibited marked attenuation of virulence. This study highlights the capacity of atmospheric pressure non-thermal plasma to modify and degrade AHL autoinducers thereby attenuating QS-dependent virulence in *P. aeruginosa*.

The ability to generate chemically rich plasmas at or near ambient temperature and their demonstrated ability to influence biological processes has led to increasing interest in their use in a range of clinical interventions, and to the emergence of the nascent field of plasma medicine. The term ‘plasma medicine’ describes the emerging multidisciplinary field of study that examines the applicability and use of atmospheric pressure non-thermal plasmas in biomedical applications and more specifically in the treatment of viable tissue. The potential uses of atmospheric plasmas in biomedical applications include skin decontamination^1^, wound treatments^2^,^3^, surface disinfection^4^,^5^ and invasive surgical treatments of tumours^6^,^7^. The physical and reactive chemistry of atmospheric pressure non-thermal plasma is derived from the production of an electric field capable of ionising air or a carrier gas such as helium/argon at atmospheric pressure. This ionisation process leads to the formation of photons, electrons, reactive species, electromagnetic energy and charged particles at a low temperature (sub 60 °C)^8^. Ambient temperatures are possible due to the inefficient transfer of energy between gas particles and electrons^9^. The efficacy of non-thermal plasma in proposed applications ranging from treatment of solid tumours, modification of cell proliferation and migration for wound healing, sterilisation and blood coagulation relies on the synergistic action of the localised generation of reactive oxygen and nitrogen species (RONS), electromagnetic energy, charged particles and UV light^10^,^11^.

Central to the development of plasma medicine, are fundamental studies between living systems (microorganisms, cells and tissue) and the reactive chemistry of atmospheric pressure non-thermal plasma^12^. Most medical applications of plasmas stem from its antimicrobial action. Studies have demonstrated tremendous efficacy against a range of clinically relevant microorganisms, in both planktonic and biofilm modes of growth^13^,^14^ and bacterial cellular targets for plasmas’ antimicrobial mode of action have been proposed^15^,^16^. Whilst the antibacterial action of non-thermal plasma is well characterised, relatively little attention has been paid to investigating the consequences of short plasma exposures on surviving microorganisms and their biomolecules. Recently Zuizana *et al.*^17^ described the potential of plasma to reduce the activity of QS controlled virulence factors in *P. aeruginosa.* However, there are, as yet, no reports of the ability of cold atmospheric plasma to directly affect quorum sensing molecules and their resulting activity in Gram negative bacteria.

Quorum sensing is utilised by bacteria to monitor cell density and co-ordinate bacterial behaviour at a community level^18^. From a clinical perspective, regulation of pathogenicity and virulence through QS-dependent mechanisms provides a strategy to ‘disarm’ pathogenic microorganisms attenuating their virulence and rendering them more susceptible to antibiotics^19^, without necessarily placing them under direct selective pressure. Bacterial QS is reliant on the production and metabolism of signalling molecules (known as autoinducers), the identity of which is distinct between Gram-positive and Gram-negative organisms. Gram-positive bacteria such as *Staphylococcus aureus* synthesise and respond to oligopeptides^20^, whereas Gram-negative bacteria primarily rely on acyl homoserine lactones (AHLs) as the primary signalling molecules involved in QS^21^. AHLs comprise a lactone ring ligated to an acyl carbon chain through an amide bond, with the acyl chain varying in carbon length, saturation or oxidation state depending on bacteria^21^. The AHL-dependent QS signalling pathways of Gram-negative bacteria rely on three main components; a gene coding for the synthase responsible for generating the AHL signalling molecule (LuxI-type gene), the signalling molecule itself and the cognate cytoplasmic receptor (LuxR-type transcription factor)^22^. This system offers three main putative targets for potential inhibition/interference of bacterial QS. *Pseudomonas aeruginosa*’s AHL-dependent QS pathways are well-characterised and provide a relevant example of QS in pathogenic bacteria. *P. aeruginosa*’s QS circuitry consists of two hierarchically-organized AHL systems (*lasIR* and *rhlIR*) which utilise AHL signalling molecules n-(3-oxododecanoyl)-homoserine lactone and n-butyryl-homoserine lactone, as well as a third 2-alkyl-4-quinolone regulated system, known as the *Pseudomonas* quinolone signal (PQS)^23^. These hierarchal systems control production of virulence factors such as elastase, pyoverdin (*las* controlled), rhamnolipid and pyocyanin (*rhl* controlled)^24^,^25^. *In vitro* and *in vivo* studies have shown reduced pathogenicity in chronic infections, through inhibition of bacterial QS^26^,^27^,^28^. Inhibition or interference of quorum sensing is thus a novel, attractive and promising strategy in the treatment of bacterial infections.

In this study, the residual activity of AHL molecules; n-butyryl-homoserine lactone (BHL), n-hexanoyl-homoserine lactone (HHL), n-octanoyl-homoserine lactone (OHL) and n-(3-oxo-dodecanoyl)-homoserine lactone (OdDHL) following plasma exposure was examined using the following QS reporter strains: *Agrobacterium tumefaciens* ATCC BAA-2240, *Escherichia coli* JM109 pSB536, pSB401 and pSB1142, and *Chromobacterium violaceum* (CV026). The double *lasI*/*rhlI* QS mutant of *P. aeruginosa*, PAO-MW1, was used to determine the capacity of plasma-exposed AHLs to restore QS-dependent virulence factor production and the virulence of *P. aeruginosa in vivo*. Figure 1 provides a schematic representation of the overall experimental design. The data presented here demonstrate, for the first time, that short plasma exposures rapidly alter both the physicochemical characteristics and the ability of AHLs to elicit QS signalling in bacterial reporter strains. Analysis of plasma-exposed AHLs by UPLC-MS and FT-IR analysis, in conjunction with plasma chemical characterization demonstrates that the interaction of plasma-derived reactive species with these molecules leads to degradation of the parent AHL, resulting in attenuation of AHL-dependent QS and virulence factor production in *P. aeruginosa*.

## Results

### Plasma exposed HHL detection using CV026

**Figure 2:**
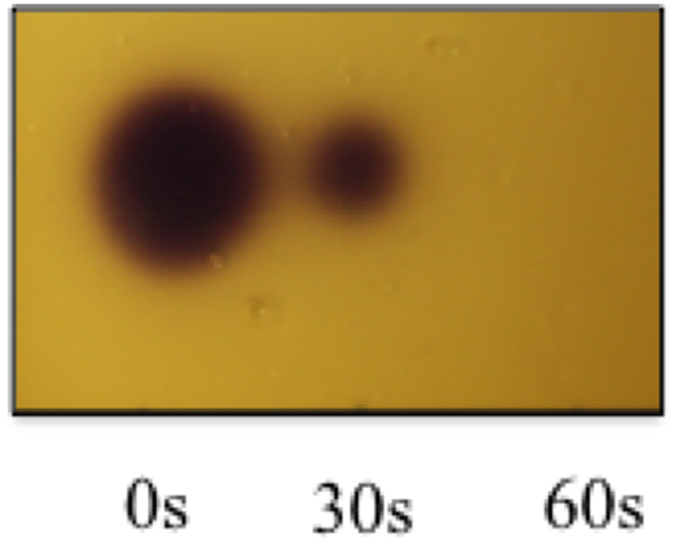
TLC-overlay of plasma treated HHL using the CV026 reporter strain. 1 μl of 0, 30, 60 seconds plasma exposed HHL (100 μM in PBS) was spotted and run using a MeOH: H_2_O (60:40) mobile phase, prior to being overlaid with molten (47 °C) LB 1% agar inoculated with CV026. Plates were incubated at 28 °C for 48 hours.

CV026 is a mini-Tn5 mutant, in which QS-dependent violacein production can be restored via the addition of exogenous AHLs with acyl side chains from four to eight carbons, with varying sensitivity^29^. QS bio-reporter strains are commonly utilized to detect AHL production by various Gram-negative bacteria^30^. The use of reporter strains to perform thin layer chromatography (TLC) -overlays of AHLs provides a simple and rapid technique used to identify AHL activity and can provide insights into the physicochemical properties of AHLs. Initially, the TLC-overlay assay was used to assess the bio-activity of plasma exposed HHL. Figure 2 displays the results of plasma exposed HHL using CV026. Following 30 seconds exposure to plasma, violacein synthesis by CV026 was clearly reduced and no observable violacein production was apparent following 60 seconds of exposure suggesting the complete inactivation/conversion of HHL.

### Detection of plasma treated BHL, HHL, OHL and OdDHL bio-activity using *A. tumefaciens* TLC-overlay

**Figure 3:**
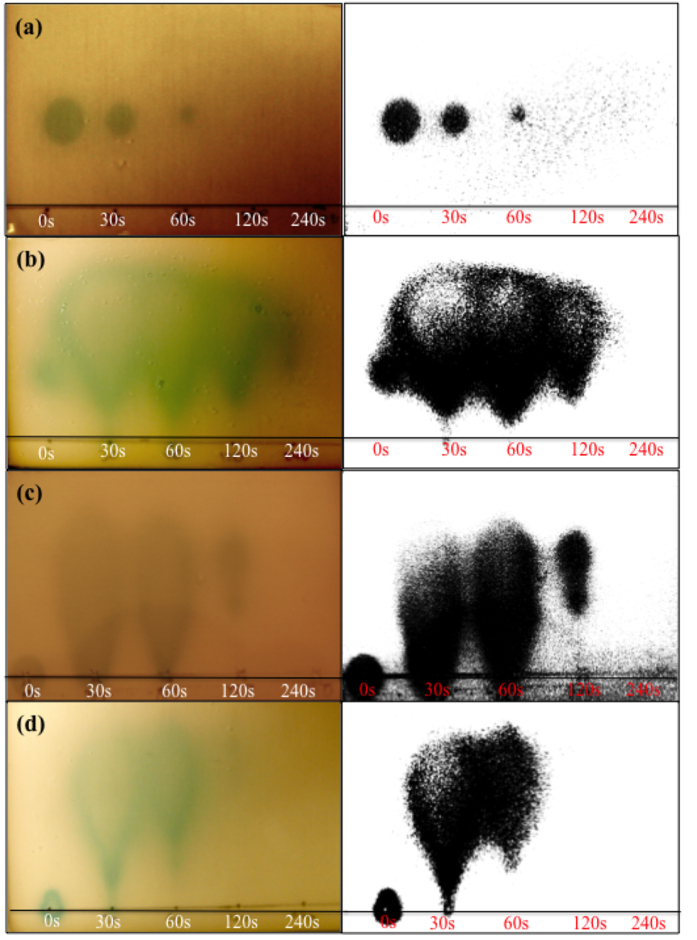
*A. tumefaciens* ATCC BAA-2240 TLC-overlay showing the effect of plasma exposure on AHL mobility and activity. (**a**) BHL, (**b**) HHL, (**c**) OHL and (**d**) OdDHL. Each AHL was exposed for 0 (positive control), 30, 60, 120 and 240 seconds. Corresponding images show original image subjected to thresholding for clarity of presentation (Image J). 1 μl of 100 μM AHL was spotted, ran and overlaid as described in materials and methods. Plates were incubated at 28 °C for 48 hours.

QS- reporter systems can be limited by the specificity for their cognate cytoplasmic receptors and AHL. The QS based receptors of these reporter strains are most efficiently activated by their cognate autoinducer, however analogues with varied chain length and oxidation/saturation state of the acyl chain may also elicit a response^31^. *A. tumefaciens* ATCC-BAA 2240 contains the *traR* gene with the fusion of the promoters *traG:lacZ*, which in the presence of exogenous AHLs synthesises β-galactosidase. This enzyme is responsible for hydrolysing the X-gal substrate, producing a blue/green colour indicative of AHL activity. *A. tumefaciens* ATCC-BAA 2240 is a sensitive and well-characterised reporter strain capable of responding to a wide variety of AHLs^31^. In fact, this reporter strain is routinely used for the detection of AHLs produced by Gram-negative bacterial isolates^32^. Due to the broader sensitivity of *A. tumefaciens* ATCC-BAA 2240, this reporter was exploited to characterise the effect of APNTP exposure on all AHLs used in this study (BHL, HHL, OHL and OdDHL). Figure 3 shows the plasma mediated effect on (a) BHL, (b) HHL, (c) OHL and (d) OdDHL using TLCs overlaid with *A. tumefaciens* ATCC-BAA 2240 alongside threshold images using ImageJ, in order to clearly identify the plasma-mediated changes to AHL activity.

Similarly to the reduction in pigment production observed when overlaying plasma-treated HHL with CV026, the overlay of treated BHL resulted in a gradual reduction in response by *A. tumefaciens* ATCC-BAA 2240 (reduced pigment zone size) with increasing exposure times. Following 120 seconds of exposure, the plasma-treated BHL was no longer capable of eliciting a visible turnover of the X-Gal substrate in this reporter. The overlay of plasma-treated HHL, OHL and OdDHL produced a different response when compared to the plasma treated BHL. In fact, there was an immediate effect on the AHL molecules after 30 seconds exposure with the generation of diffuse comet shaped zones. These diffuse comet shaped response zones covered a larger area than the control, and when compared to the untreated controls, an extensive migration of the plasma treated HHL, OHL and OdDHL was observed. With increasing plasma exposure the pigment zones decreased in size for HHL, OHL, OdDHL, with no detectable activity for OHL and OdDHL after 240 and 120 seconds plasma exposure respectively. Plasma treated HHL was still capable of eliciting a visible response in the *A. tumefaciens* ATCC-BAA 2240 reporter even after 240 seconds of exposure to plasma highlighting the differences in sensitivity when compared to the CV026 reporter. However, the response zone obtained from 240 seconds plasma treated HHL overlaid with *A. tumefaciens* ATCC-BAA is smaller compared to the untreated control, suggesting a gradual inactivation over increasing exposure times. The plasma treated HHL also appears to have a different migration suggesting either the oxidation of the acyl side chain or chain shortening effects. Lactone ring opening would be expected to result in complete loss of pigment production and may be responsible for the loss of signal at longer AHL exposure times.

### Quantification of the bioactivity of plasma exposed HHL, OHL and OdDHL using luminescence-based reporters *E. coli* strains pSB 536, 401 and 1142

**Figure 4:**
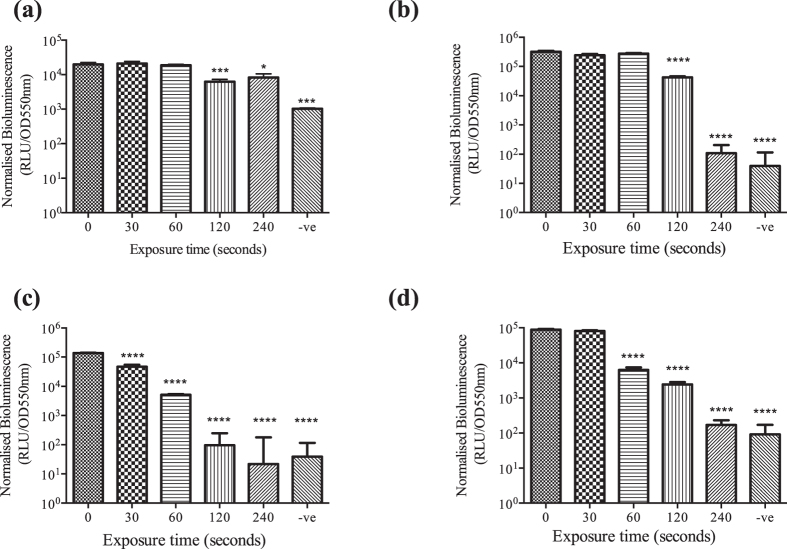
Normalised bioluminescence after 5 hours growth for bioluminescence reporters *E. coli* pSB536 (**a**) BHL (10 μM) incubated at 30 °C, *E. coli* pSB401 for plasma treated (**b**) HHL (10 μM), (**c**) OHL (10 μM) and *E.coli* pSB1142 for plasma treated (**d**) OdDHL (10 μM) incubated at (37 °C) for 14 hours. Bioluminescence plateaued after 4 hours, increasing proportionally to growth of *E. coli* (S1) Each AHL was plasma treated for 0, 30, 60, 120, 240 seconds and reporter strain only (−ve). Error bars represent the standard deviation of five replicates. One way ANOVA with post hoc Dunnett’s multiple comparisons test compared to 0 seconds was used for statistical analysis. ****p value < *0.0001.*

Bioluminescence assays using *E. coli* strains pSB536 (BHL), pSB401 (HHL, OHL) and pSB1142 (OdDHL) reporter strains were used to quantify the residual bioactivity of plasma-treated BHL, HHL, OHL and OdDHL. *E. coli* pSB536 contains the *ahyR ahyI::luxCDABE* which responds to short chain AHLs primarily BHL. *E. coli* JM109 pSB401 is a recombinant based AHL biosensor, were the *luxR* and *lux* promoter region from *Photobacterium fischeri* is coupled to the entire *lux* operon (*LuxCDABE)* from *Photorhabdus luminescenc*s^33^. This construct, when expressed in *E. coli*, responds to medium chain AHLs (C6-C8) producing bioluminescence. *E. coli* JM109 pSB1142 is a long-chain reporter carrying the *las*R and *las* promoter of *P. aeruginosa* fused to the *luxCDABE* cassette from *Photorhabdus luminescens* responding to long-chain AHLs (C10-C14). To exclude growth-dependent effects, OD measurements were recorded in order to normalize bioluminescence production to cell density. Figure 4 shows normalized bioluminescence results for (a) BHL, (b) HHL, (c) OHL and (d) OdDHL after 5 hours incubation. This time point was chosen from bioluminescent growth curves obtained over 14 hours found as Supplementary Information Fig. S1. Figure 4 depicts normalised bioluminescence at 5 hours in order to scrutinise the effect of plasma exposure on each AHL. A negative (−ve) control containing PBS and the reporter strain was used as a blank control and considered as a “nil response”. Figure 4(a) displays the bioluminescence of plasma exposed BHL using reporter strain pSB536. No decrease in bioluminescence was observed following 30 and 60 seconds exposure. A significant decrease in bioluminescence was observed following 120 seconds exposure (*p* < *0.0001*). However, following 120 seconds and 240 seconds exposures of BHL bioluminescence was still detectable in pSB536, when compared to the negative control. A significant (*p* < *0.0001*) decrease in luminescence was observed after 120 seconds plasma exposure of HHL (Fig. 4(b)) comparable to the blank control. Figure 4(c) displays the effect of plasma pre-exposure of OHL on QS-dependent bioluminescence; 30 seconds exposure is sufficient to cause a significant (*p* < *0.0001)* decrease in bioluminescence whereas 240 seconds of plasma exposure of OHL gave rise to normalized luminescence values equivalent to the blank control. Increasing plasma exposure of OdDHL Fig. 4(d) resulted in a decrease in bioluminescence of *E. coli* JM109 pSB1142 and a significant reduction after 60 seconds exposure with a decrease in bioluminescence with increasing plasma exposure.

### Effect of plasma exposed BHL and OdDHL on QS-dependent virulence factor production using the *las*I/*rhl*I double mutant PAO-MW1

**Figure 5:**
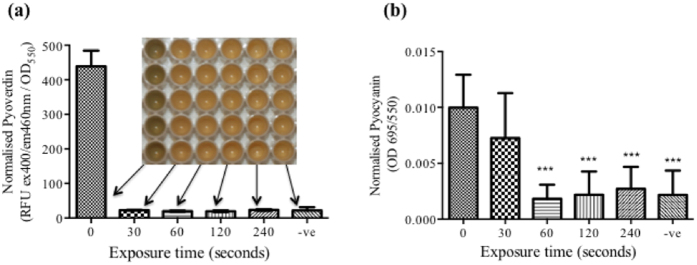
Pyoverdin and pyocyanin production by PAO-MW1 incubated (37 °C) with BHL and OdDHL (10 μM final concentration). AHLs were exposed to plasma for 0, 30, 60, 120 and 240 seconds, reporter strain only (−ve). (**a**) Normalised pyoverdin production following 24 hours, with corresponding plate were fluorescent measurements were taken. (**b**) Normalised pyocyanin production after 24 hours incubation (37 °C) with plasma exposed BHL, OdDHL and PAO-MW1. Error bars represent the standard deviation of five replicates. One way ANOVA with post hoc Dunnett’s multiple comparisons test compared to 0 seconds, untreated AHLs were used for statistical analysis. ***p value < *0.001*.

In order to investigate the effect of plasma exposure of BHL and OdDHL on virulence factor production *in vitro*, a *las*I/*rhl*I double QS mutant of PAO1 (PAO-MW1) was incubated with the plasma exposed AHLs. The PAO-MW1 double mutant was generated through insertional mutagenesis of *las*I into the *rhl*I deleted mutant PD100^25^,^34^. This mutant is unable to synthesise the two cognate AHL autoinducers BHL and OdDHL produced by wild type *P. aeruginosa*, although the transcriptional activators *las*R and *rhl*R are still active. This allows restoration of the expression of AHL-dependent genes when BHL and OdDHL are added exogenously. Cultures were incubated for 24 hours at 37 °C. After 24 hours, plates were read at OD_550_ to normalize pyoverdin and pyocyanin production to cell density. Pyoverdin is a fluorescent siderophore which can be measured at ex400/em460 nm^35^. For pyocyanin measurements, the absorbance of culture supernatants was read at a wavelength of 695 nm^36^. Figure 5(a) exhibits pyoverdin production of PAO-MW1 following the addition of plasma exposed BHL and OdDHL. The inlaid image above the bar chart depicts the setup of the 96-well plate used to perform the readings. The presence of pyoverdin is distinguishable from the green colour of the positive control inoculum. PAO-MW1 grown in the presence of BHL and OdDHL treated with plasma for 30 seconds was unable to synthesise pyoverdin compared to incubation with the untreated BHL and OdDHL controls. Figure 5(b) shows normalized pyocyanin production by PAO-MW1 incubated with both BHL and OdDHL. Incubation of PAO-MW1 with BHL and OdDHL treated for 30 seconds caused a significant decrease in pyocyanin production. No pyocyanin production was obtained when PAO-MW1 was incubated with BHL and OdDHL exposed for 60 seconds or more.

### *In vivo* assessment of plasma exposed BHL and OdDHL capacity to induce virulence in PAO-MW1

**Figure 6:**
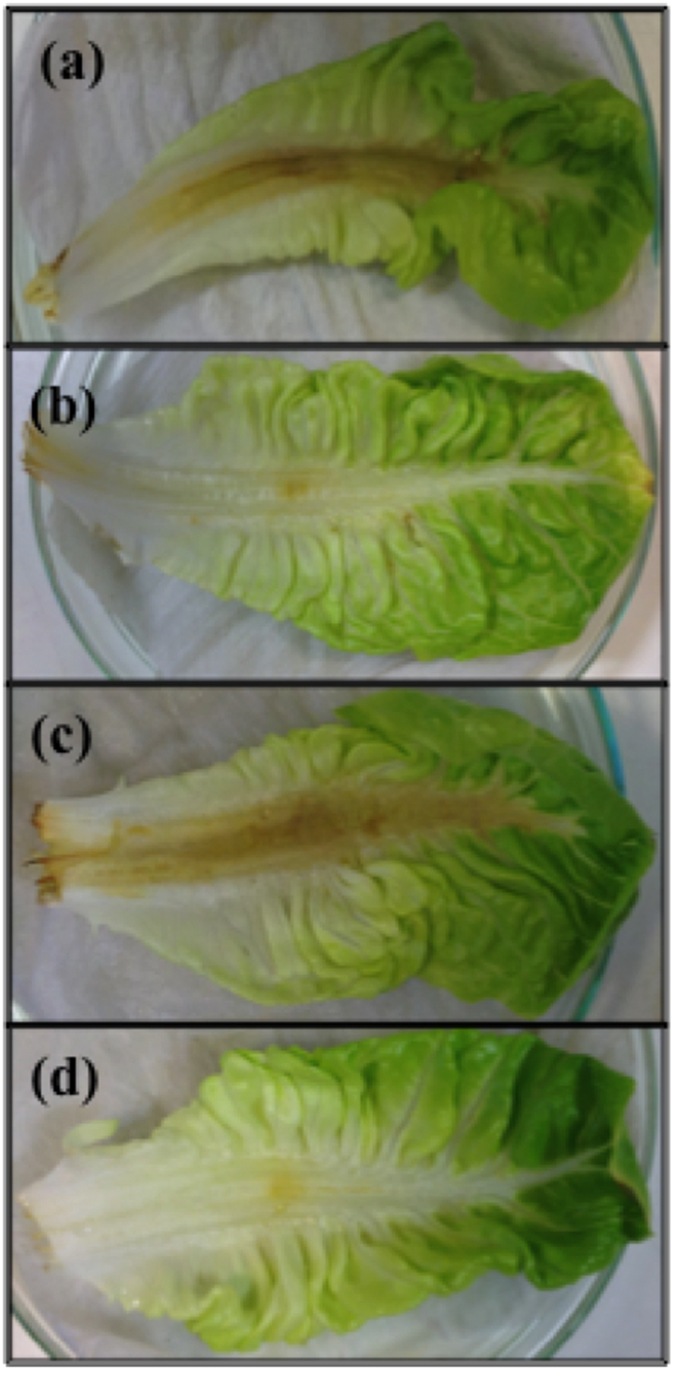
Lettuce leaves after three days incubation with (**a**) PAO1, (**b**) PAO-MW1 only (**c**) PAO-MW1 with 10 μM BHL: OdDHL and (**d**) PAO-MW1 with 10 μM of 120 seconds plasma treated BHL: OdDHL. 10 μl of a 10^7^ CFU/ml bacterial suspension was injected into the mid-rib of five lettuce leaves for each condition with leaves monitored daily for three days at 37 °C.

The lettuce leaf model is a standard, cost-effective and simple measure of bacterial virulence, eliminating the regulations and ethical considerations associated with animal models^37^. It has previously been demonstrated that animals and plants share common virulence factors^38^ allowing the use of such a model for the measurement of virulence. Images shown in Fig. 6 allows visual inspection of the virulence of PAO-MW1. Brown discoloration of the lettuce stem indicates necrosis induced by the bacteria. By comparing (a) PAO1 the wild type control, (b) PAO-MW1 only and (c) PAO-MW1 & 10 μm of BHL: OdDHL, the effect of AHLs on the *in vivo* virulence of *P. aeruginosa* is apparent. Figure 6(b) shows a limited amount of discolouration at the site of injection with 6 (c) showing diffuse necrosis and discolouration along the full length of the stem comparable to the wild type PAO1 (Fig. 6(a)). Figure 6(d) depicts the virulence of PAO-MW1 combined with plasma treated BHL and OdDHL. The discoloration at the injection site is comparable to that of PAO-MW1 control (Fig. 6(b)) and can be clearly appreciated that the plasma treated AHLs are unable to restore the virulence of *P. aeruginosa* shown in Fig. 6(c).

### FT-IR analysis and UHPLC-MS

**Figure 7:**
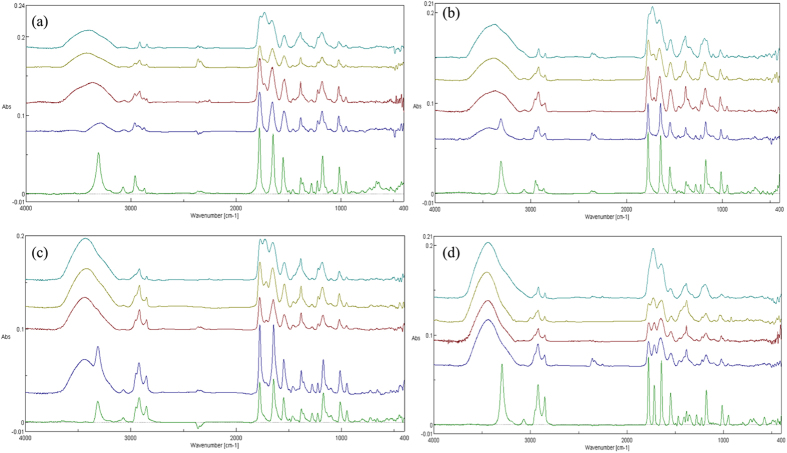
FT-IR spectra of (**a**) BHL, (**b**) HHL, (**c**) OHL, (**d**) OdDHL dried onto KBr discs, following plasma treatment. Untreated (green line), 30 seconds exposure (blue line), 60 seconds exposure (red line), 120 seconds (yellow), 240 seconds (turquoise).

**Figure 8:**
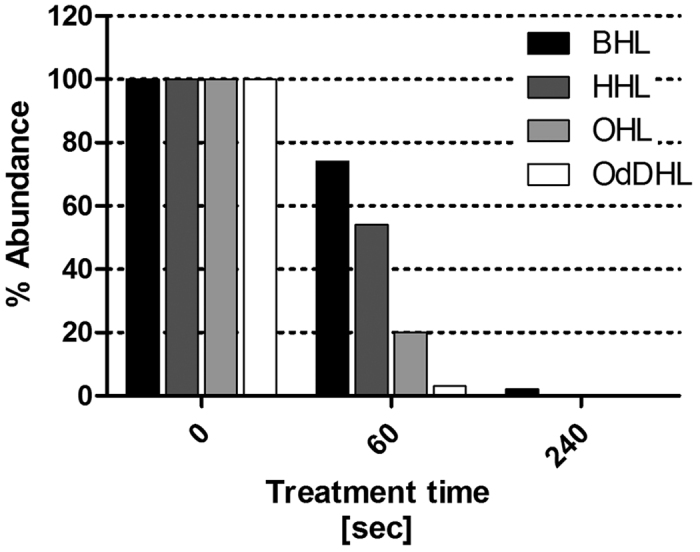
Plasma exposure time-dependent degradation of AHL parent molecules; abundance of the analytes peaks following plasma treatment based on the UHPLC-MS analysis. The bars represent the absolute response of the analyte, measured as peak area.

For FT-IR and UHPLC-MS analysis the concentration of AHLs exposed to the plasma was increased to 1 mM. To negate any influence of PBS components, deionised water was used as the sample medium for plasma exposure. The change from PBS to water did not alter the physicochemical changes observed with plasma exposure of AHLs in PBS seen with Fig. S7 in supplementary material. Figure 7 displays FT-IR spectra for all four AHLs with plasma treatment for each plasma exposure time offset. Samples were freeze dried after plasma treatment in order to remove water with the resultant product solubilised in acetonitrile and dried onto KBr discs. FT-IR analysis clearly shows presence of an OH functional group as a result of plasma exposure. Moreover, a time dependent production of a new C=O peak at approximately 1720 cm^−1^ is also apparent. UHPLC-MS analysis shows an exposure time-dependent decomposition of the original AHLs with a significant reduction in the concentration of the four AHLs used in this study following just 60 seconds exposure, and with the exception of BHL, the complete eradication of the original molecules following 240 seconds exposure (Fig. 8). The UHPLC-MS analysis highlighted a difference in the susceptibility to plasma-induced decomposition (caused by plasma exposure) dependent on the length of the acyl side chain of the AHL autoinducer molecules. Shorter chain AHLs were found to resist degradation more than longer chain AHLs.

## Discussion

The well-characterised, multispecies antimicrobial activity of atmospheric pressure non-thermal plasma is naturally at the basis of most studies investigating the potential use of plasma in a variety of medical settings and applications^3^,^39^. Recently, the use of cold plasma for the topical treatment of chronic wounds has been proposed. The therapeutic potential of such an approach could eventually provide both an antimicrobial treatment to prevent or treat acute and chronic wound infections whilst stimulating wound healing and tissue regeneration^40^. To date no studies have investigated the effects of short exposures (where complete eradication is not achieved) of non-thermal plasma on bacterial QS directly, a crucial regulatory mechanism utilised by numerous bacterial pathogens to regulate virulence and biofilm formation^22^. In this study, a panel of QS bio-reporter strains capable of responding to the acyl homoserine lactone (AHL) signalling molecules, alongside chemical analysis (FTIR analysis and UHPLC-MS) were used to interrogate the effects atmospheric plasma treatment has on BHL, HHL, OHL and OdDHL signalling molecules. Plasma exposed AHLs were examined for their residual capacity to elicit a QS-dependent response *in vitro* as well as their capacity to control virulence of *P. aeruginosa* using a lettuce leaf virulence model. FTIR and UHPLC-MS analysis provided structural insights into the plasma-induced modification of these molecules which, when combined with the physical and chemical characterization of the plasma and of the ROS produced by the plasma, provides valuable insight into the reactive species responsible for the structural effects observed.

The TLC overlay assay of plasma-exposed HHL using the CV026 reporter strain (Fig. 3) suggests the gradual inactivation and loss in bioactivity of HHL following just 60 seconds exposure to atmospheric plasma. Interestingly, the TLC overlay assay of plasma-exposed, HHL, OHL and OdDHL using the *A. tumefaciens* ATCC-BAA 2240 bio-reporter (Fig. 4), demonstrated the presence of AHL active components with different physicochemical properties produced by plasma exposure. These results suggest the occurrence of a complex series of chemical modifications following plasma exposure, which were still capable of eliciting a QS-dependent response with the bio-reporter. In fact, when plasma-exposed HHL was run on TLC and overlaid with *A. tumefaciens* ATCC-BAA 2240, the reporter strain detected and responded to the exposed AHLs generating diffuse comet-like zones of pigment production with some residual activity still apparent following 240 seconds of plasma exposure. Whereas the bio-reporter CV026 readily responds to low concentrations of HHL, it is less sensitive to BHL and OHL and requires much higher concentrations to elicit violacein biosynthesis^29^. The difference in sensitivity of these bio-reporters could explain the inconsistencies observed when comparing the results of the two assays for HHL. In fact, *A. tumefaciens* ATCC-BAA 2240 responds to a wider range of AHLs than CV026 and at much lower concentrations including 3-oxo and 3-hydroxy derivatives of homoserine lactones^30^. The latter observation indicates that CV026 was unable to respond to the plasma induced changes to HHL that *A. tumefaciens* ATCC-BAA 2240 continued to respond to up to 240 seconds. Whilst CV026 appeared to indicate a complete inactivation of the AHLs signalling potential, the *A. tumefaciens* ATCC-BAA 2240 bio-reporter was capable of responding to the AHL products produced by plasma pre-exposure.

The diffuse elliptical zones of pigment production observed on the TLC overlaid with *A. tumefaciens* ATCC-BAA 2240 of HHL, OHL and OdDHL following plasma treatment (Fig. 3) are characteristic and indicative of the presence of 3-oxo derivatives^31^. In fact, following 30 seconds of plasma exposure the zones of pigment production generated by the plasma-treated AHLs change significantly in shape and distribution becoming larger, more diffuse and comet-shaped when compared to the zones of activity of the control HHL and OHL and the small elliptical zone produced by OdDHL. These results show that atmospheric plasma is capable of altering the physicochemical characteristics of these AHLs. In particular, plasma-exposed OHL and OdDHL migrated further on the RP-TLC plate indicating plasma treatment has rendered the treated molecules more hydrophilic, possibly through the addition of polar substituents, an observation which is in keeping with the presence of the OH functional group stretch shown in Fig. 7 for all plasma treated AHLs. Plasma characterization data presented in Figs S2–S4 describe the reactive species produced in the plasma gas phase and plasma treated water and PBS. Figures S3 and S4 demonstrate the production of 2-hydroxyterepthalic acid and pertitanic acid as indicators for the production of hydroxyl radicals and hydrogen peroxide respectively. AHLs were incubated with 4 mM hydrogen peroxide for four minutes (equivalent to the hydrogen peroxide concentration produced by plasma after 240 s exposure) and overlaid using the *A. tumefaciens* assay. Figure S8 demonstrates that hydrogen peroxide is not responsible for the physicochemical effects observed following plasma exposure of AHL molecules, as described in this study. Although plasma exposure of aqueous media produces a wide range of reactive specues^41^,^42^,^43^, the presence of an OH functional group in plasma treated AHL samples seen with FT-IR analysis correlates with the production of the fluorescent 2-hydroxyterephtalic acid from the non-fluorescent terephthalic acid, indicating the importance of the hydroxyl radical produced by the plasma as one of the reactive species responsible for the effects demonstrated. Work by Frey *et al.* supports this finding where they investigated the effect on signalling activity of AHLs treated with the OH radical specifically, reporting that the reaction between HHL and OHL produced several species characterised by oxidation and hydroxylation of the acyl chain^44^.

Whilst the overlay of plasma exposed HHL, OHL and OdDHL using the reporter strain *Agrobacterium tumefaciens* ATCC-BAA2240 produced comet-like spots, plasma exposure of BHL did not give rise to this phenomenon. However the addition of OH functional groups (deduced from FT-IR) indicate a limitation in the sensitivity of the *A. tumefaciens* assay in detecting hydroxyl or oxo derivatives of BHL, in accordance to previously reported data^31^. In contrast, the plasma treatment of BHL resulted in a reduction in response zone size with increasing exposure times and total loss of bio-activity following 60 seconds of exposure. This chain-length dependent effect was shown using the *A. tumefaciens* assay and confirmed with UHPLC-MS analysis (Fig. 8, Figs S9–S10). As longer acyl side chains donate electrons more readily stabilizing AHLs against lactone ring opening^41^, the differences in chain length of BHL, HHL, OHL and OdDHL are also important in dictating the nature of the modifications during plasma exposure. Side chain modifications of the longer chain AHLs which are more easily oxidised occur more readily and would explain the chain length dependent decomposition of the parent AHL molecule. BHL has a short (four carbon) acyl side chain and may not be as susceptible to chain modification as AHLs with longer acyl side chains. Modifications of the lactone ring of the molecule, with possible ring opening may account for the attenuation of QS-dependent pigment production. FT-IR analysis of the lactone stretch at 1775 cm^−1^ representative of the C=O present on the lactone ring is shown to gradually decrease with plasma exposure for all AHLs and its disappearance after 240 seconds exposure of OdDHL coincides with the loss of visible QS induced response, as seen in the supplementary information Figure S7 for 1 mM plasma exposed OdDHL in PBS and water. It could then be expected that the comet like zones shown with the AHLs HHL, OHL and OdDHL may well be a combination of plasma modified AHLs with hydroxyl and oxo derivatives with varying polarities accounting. The relation between the formation of comet- like zones of response observed in the TLC overlay assays and the presence of hydroxyl and oxo derivatives on the acyl side chain is currently being investigated using UHPLC-MS analysis (Fig. S10). This approach has highlighted the limitation of FT-IR analysis as whilst information can be garnered based on the presence of functional groups such as the OH group and the time dependent increase in new stretch at C=O 1720 cm^−1^ it cannot distinguish between the formation of a multitude of products, supposedly responsible for the peak broadening displayed with increasing plasma exposure.

The results of the TLC-overlay using *A. tumefaciens* ATCC-BAA 2240, FT-IR and UHPLC-MS data following plasma exposure of the longer chain AHLs (HHL, OHL, OdDHL) suggests atmospheric plasma may readily oxidise the acyl chains. This offers an explanation for the formation of the comet-like spots and the gradual loss of bioactivity of HHL, OHL OdDHL observed using *A. tumefaciens* ATCC-BAA 2240 (Fig. 2) and confirmed in the luminescence assay (Fig. 3) when using the reporter strains pSB401 and pSB1142. As well as *A. tumefaciens* ATCC-BAA 2240, the luminescent based reporters contain dynamic promoters capable of responding to different analogues of AHLs of the same chain length^33^, such as those produced by plasma exposure. Ultimately, the findings from the UHPLC-MS and the TLC overlays indicating the presence of a series of AHL by-products requires further work involving the purification and structural elucidation of the products.

Considering the potential use of cold plasmas, for example, in the topical treatment of wounds, the putative production of modified, active AHLs following short plasma exposures of native signalling molecules is of crucial importance, and requires further investigation both from a biological and clinical perspective. Therefore we investigated the effect of plasma exposure of QS molecules on the virulence and pathogenicity of *P. aeruginosa*. This common problematic pathogen is often found in association with burn-wound victims and type II diabetes-related chronic ulcers and wounds^42^,^43^, an area where atmospheric pressure non-thermal plasma is likely to find rapid clinical translational application^9^. Figure 5 shows virulence factor production from a double *lasI/RhlI* QS mutant that is unable to synthesise its two cognate AHL signalling molecules BHL and OdDHL. Plasma exposure of the relevant AHL molecules, BHL and OdDHL were unable to restore pyoverdin production at any of the plasma exposure time points, suggesting that the modifications resulting from plasma exposure of OdDHL are unable to induce a response in the *las* system. After 30 seconds exposure there was a significant reduction, equivalent to the negative control for pyocyanin production. BHL is the signalling molecule used by the *rhl* QS system and is responsible for controlling the production of pyocyanin. The production of pyocyanin correlates with the decreasing size of spots in the *A. tumefaciens* assay suggesting that BHL is not extensively modified compared to OdDHL, and that the readily modified plasma treated OdDHL cannot induce AHL dependent QS in *P. aeruginosa.* Whilst understanding the role plasma treated BHL and OdDHL have on the production of individual virulence factors *in vitro*, we have also demonstrated that this response is translated *in vivo* using a lettuce leaf virulence model. Figure 6 shows lettuce leaves injected with the double mutant PAO-MW1 in the presence or absence of plasma treated AHLs alongside the untreated molecules. It has been demonstrated that AHLs are important for *P. aeruginosa* virulence and herein we demonstrate for the first time that the modification of AHLs, observed using the *A. tumefaciens* reporter assay do not restore the virulence of *P. aeruginosa.* Plasma exposure of AHL signalling molecules therefore has the potential to reduce QS-regulated virulence and pathogenicity *in vivo* at short exposures.

## Conclusion

The results illustrated in this study demonstrate for the first time that, beyond the well characterised antimicrobial effects of plasma exposure, atmospheric pressure non-thermal plasma can be used to attenuate bacterial quorum sensing and interfere with QS dependent virulence factor production directly in Gram-negative bacteria. Using microbiological bio-reporters and detailed chemical analysis, we demonstrate that AHL molecules are altered rapidly on plasma exposure, giving rise to a AHL derivatives and, ultimately, complete attenuation and inhibition of signalling. This study for the first time offers insights into how plasma exposure can rapidly attenuate QS dependent virulence factor production as well as providing insights into the mechanistic interaction of atmospheric plasma and AHL molecules, leading to chemical modification and degradation of the parent molecules. Whilst further experimental analysis of the plasma treated AHLs is currently underway in order to gain a greater understanding and knowledge of these complex interaction, these data provide necessary and pertinent information for the application of cold plasma in clinical applications, where ultimately the complex interplay between plasma generated reactive species and biological molecules must be given appropriate consideration.

## Materials and Methods

### Reporter strains and Growth conditions

*Pseudomonas aeruginosa* PAO1 (ATCC BAA-47) was obtained from the American Type Culture Collection and cultured in LBB at 37 °C. The bacterial QS-reporter strains used in this study are *Chromobacterium violaceum* CV026 (cultured in Luria Bertani broth (LBB) at 28 °C and maintained in kanamycin 25 μg/mL), *Escherichia coli* JM109 pSB 536 (Cultured in LBB at 30 °C maintained in ampicillin 25 μg/mL) *E. coli* JM109 pSB401 (cultured in LBB broth at 37 °C and maintained in tetracycline 20 μg/mL), *E. coli* JM109 pSB1142 (cultured LBB at 37 °C and maintained in tetracycline 20 μg/mL), *Agrobacterium tumefaciens* ATCC BAA-2240 (cultured in LBB at 28 °C and maintained in gentamicin 15 μg/mL) and *Pseudomonas aeruginosa* PAO-MW1 (cultured in LBB at 37 °C and maintained in tetracycline 50 μg/mL and mercury chloride 15 μg/mL). A summary of the bioreporters strains used in this study and their source is found as Supplementary Table S1.

### Atmospheric Pressure Non-Thermal Plasma Source and Treatment Conditions

The atmospheric pressure non-thermal plasma source used in this study was developed in-house at the School of Plasma Physics, Queens University Belfast, and previously described in detail^14^,^45^,^46^. The exposure of AHL solutions to plasma was conducted using the plasma source operating with a 2 Standard Litres per Minute (SLM) flow of a mixture of helium and oxygen (0.5%) and the output of a high voltage pulse source (Haiden PHK-2k) at a voltage amplitude of 6 kV and a repetition frequency of 20 kHz. For AHL exposure experiments, a sample of 20 μl of a 100 μM AHL solution in phosphate buffer saline (PBS) or deionised water was exposed to the plasma effluent at atmospheric pressure at a distance of 15 mm from the nozzle of the quartz plasma source. AHL sample solutions were exposed for 0, 30, 60, 120 and 240 seconds. Five replicates of each sample were exposed per time point. The effect of plasma exposure on the pH (PBS and water) and temperature (PBS) was also examined (See Supplementary Materials and Figs S5 and S6). To take into account the effect of evaporation on the concentration of test solutions, following plasma exposure the volume of test-solutions was re-adjusted to 20 μl by adding an adequate volume of PBS (based on the weight of test solutions determined using a microbalance before and after plasma exposure).

### Acyl homoserine lactones

A 10 mM stock solution of BHL, HHL, OHL and OdDHL (Sigma Aldrich, Dorset, UK) in acetonitrile was diluted in phosphate buffer saline (PBS) to achieve a 100 μM working concentration of each AHL used for plasma exposure. All stock solutions were either used immediately or stored at −20 °C.

### Thin layer Chromatography (TLC) overlays

The TLC overlay assays using reporter strains *C. violaceum* CV026 and *A. tumefaciens* ATCC BAA-2240 were conducted as previously described by Yates *et al.*^41^, with slight modifications. Following exposure to plasma, 1 μl of exposed AHL solutions (100 μM) and 1 μl of the relevant untreated AHL controls (100 μM) were spotted on RP-C18 TLC plates (Merck Ltd, Germany), developed using a MeOH: H_2_O (60:40 v/v) mobile phase, air-dried in a class 2 laminar flow microbiology cabinet, placed in empty sterile petri dishes and then overlaid with molten LBA (1%) seeded with overnight cultures of relevant reporter strains. Bacterial overlays using CV026 were performed by seeding 10 ml of molten (47 °C) LB + 1% agar with 50 μl of an overnight culture of CV026. Bacterial overlays using *A. tumefaciens* ATCC BAA-2240 were performed by seeding 10 ml of molten (47 °C) LB + 1% agar with 1 ml of an overnight culture of *A. tumefaciens* ATCC BAA-2240 with a final concentration of 60 μg/ml X-gal (5-Bromo-4-chloro-indoyl β-D-galactoside). Overlaid plates were left at room temperature to set for two hours before incubation at 28 °C for 48 hours.

### Bioluminescence assay

The reporter strains *E. coli* JM109 pSB536 (short chain C4 homoserine lactones (HSL)) *E. coli* JM109 pSB401 (medium-chain (C6-C8 HSL) biosensor) and JM109 pSB1142 (long-chain (C10-C12 HSL) biosensor) were used to measure luminescence in response to incubation with plasma-treated BHL (pSB536), HHL, OHL (pSB401) and OdDHL (pSB1142). Reporter strains were inoculated directly from frozen stocks into 100 ml of LBB with the appropriate antibiotic and incubated overnight.

10 μl of plasma exposed AHL samples (100 μM) and 90 μl of a 1 in 100 dilution of an overnight culture of *E. coli* pSB 536/pSB401/pSB 1142 (where appropriate for each AHL) were placed in each well of a Nunc 96-well plates (Fisher Scientific UK Ltd., Loughborough, UK) resulting in a final AHL concentration of 10 μM. 10 μl of PBS was added in place of AHLs as a negative control (−ve). 10 μl of untreated AHL samples (100 μM) were added to 90 μl of inoculum as a positive control. Five replicate wells were used for each test condition/control. Luminescence was measured over 14 hours at 37 °C and 30 °C for pSB536 using a BMG Fluostar Optima Fluorescence plate reader (BMG Labtech Ltd, Aylesbury, UK). Values were normalized according to growth (OD_550_) over the same period for each of the five replicates using a microplate reader (BioTek EL808; BioTek Instruments Ltd., Potton, UK). Luminescence curves were constructed by plotting the logarithmic normalized bioluminescence versus the incubation time and are displayed in Supplementary Fig. S1.

### Pyoverdin and Pyocyanin production of PAO-MW1

BHL and OdDHL were added to PAO-MW1 at a 1:1 ratio, with a final concentration of 10 μM. 20 μl of both plasma exposed BHL and OdDHL were added to 160 μl of a 1 in 100 dilution of an overnight culture of PAO-MW1 in LBB or *Pseudomonas* broth for the pyocyanin assay into a Nunc 96-well plate (Fisher Scientific UK Ltd., Loughborough, UK). Cultures were incubated for 24 hours at 37 °C. Pyoverdin production was measured using a fluorescence plate reader^35^ (BMG Labtech Ltd., Aylesbury, UK) (ex 400 nm, em 460 nm). Pyocyanin was measured at OD_695_ from the supernatants of overnight cultures using a microplate reader^47^ (BioTek EL808; BioTek Instruments Ltd., Potton, UK). Pyocyanin and Pyoverdin values were normalized according to growth at OD_550_ after 24 hours growth. PBS added to cultures instead of AHLs was used as a negative control (−ve).

### Lettuce Leaf Infection Model

The lettuce leaf infection model based on Starkey *et al.*^37^ protocol was used to qualitatively describe the effect plasma treated AHLs have on the *in vivo* virulence of *P. aeruginosa*. Romaine lettuces were bought from a commercial supermarket. The outer leaves of the lettuce were discarded with the remaining leaves stripped and washed in 0.1% bleach and rinsed twice with sterile water. The stripped leaves were injected with 10 μl of a 10^7^ CFU/ml PAO-MW1 and PAO1 in LBB suspension that had been incubated for four hours at 37 °C with either 120 second plasma treated BHL: OdDHL or no plasma treated BHL: OdDHL at a final concentration of 10 μM. PAO1 was incubated with PBS only. After inoculation, the leaves were incubated at 37 °C in glass petri dishes on PBS soaked tissue paper for three days. Five lettuce leaves were used for each condition and each condition was repeated on three separate occasions.

### FTIR analysis of plasma treated AHLs

After plasma exposure of AHLs, BHL, HHL, OHL and OdDHL samples were frozen and freeze dried. Once freeze dried, AHLs were solubilised with 20 μl of acetonitrile and spotted onto FTIR grade potassium bromide discs (Sigma Aldrich, Dorset, UK) for analysis. FTIR measurements were performed on a Jasco 4000 series FTIR (Jasco Inc. Tokyo, Japan) with spectra collected from 4000–400 cm^−1^ with a resolution of 4 cm^−1^ and an average of 64 scans, blank measurements were taken between each sample and FTIR spectra of plasma exposure of AHL’s taken on three separate occasions.

### Targeted Analysis

Separations were performed using a Waters, Corp. (Milford MA, USA) Acquity UPLC I-Class system comprising of a stainless steel Kinetex® F5 analytical column (2.1 × 100 mm, particle size 1.7 μm) from Phenomenex, Inc. (Torrance, CA, USA) maintained at a temperature of 55 °C while the pump was operated at a flow rate of 0.4 mL/min. A binary gradient system was used to separate analytes comprising of mobile phase A, 0.1% (v/v) formic acid in H_2_O and mobile phase B, MeOH. The gradient profile was linear from 99% A to 1% over 10 min and held for 1 min, followed by 3 min for re-equilibration at 99% A. Typical chromatogram is presented in Fig. S9.

Analytes were detected using a Waters, Corp. (Milford, MA, USA). TQ-S quadrupole instrument operating in positive electrospray ionisation (ESI) mode (more detailed conditions are presented in Table S2). The UHPLC - MS system was controlled by MassLynx™ software and data was processed using TargetLynx™ software (both from Waters). The electrospray voltage was set at 2.5 kV, the desolvation and source temperatures were set at 500 and 130 °C, respectively. Nitrogen was employed as the desolvation and cone gas and its flow rate was set to 700 L/h and 150 L/h, respectively. Argon was used as the collision gas, at a flow rate of 0.15 mL/min, which typically gave pressures of 2.3 × 10^−3^ mBar. Samples were reconstituted in H2O:MeOH (9:1, v/v) and 1 μL injected onto the system.

### Statistical Analysis

Statistical analyses were performed using GraphPad Prism 6 (GraphPad Software Inc., San Diego, CA). The means and standard deviations for the bioluminescence assays and virulence factor production (pyoverdin and pyocyanin) assays were calculated based on five replicates. Parametric one way Analysis of Variance (ANOVA) was used to analyze statistical differences with Dunnett’s *Post hoc* analysis for bioluminescence and virulence factor production. Data was shown to be normally distributed using the Kolmogorov and Smirnov method. In all cases, a probability of *p* < *0.05* (*) denoted significance.

## Additional Information

**How to cite this article**: Flynn, P. B. *et al.* Non-thermal Plasma Exposure Rapidly Attenuates Bacterial AHL-Dependent Quorum Sensing and Virulence. *Sci. Rep.* **6**, 26320; doi: 10.1038/srep26320 (2016).

## Supplementary Material

Supplementary Information

## Figures and Tables

**Figure 1:**
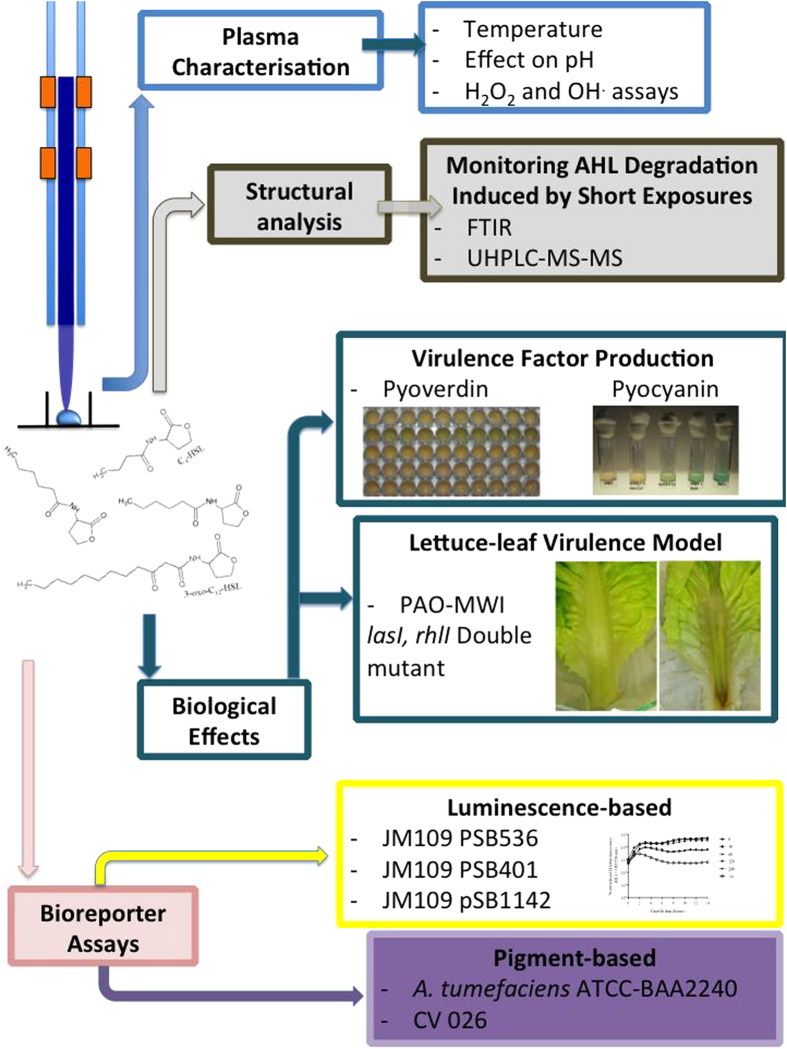
Schematic diagram illustrating the strategies adopted to determine the effect of plasma exposure on AHLs.
