# Supplementary material for: Non-thermal Plasma Exposure Rapidly Attenuates Bacterial AHL-Dependent Quorum Sensing and Virulence
Source: Sci Rep. 2016 May 31;6:26320. doi: 10.1038/srep26320 (PMC4886528; doi:10.1038/srep26320)
Supplement: Supplementary Information [file srep26320-s1.docx]

**Supplementary Information: Non-thermal Plasma Exposure Rapidly Attenuates Bacterial AHL-Dependent Quorum Sensing and Virulence**

Padrig B. Flynn^1,2,†^, Alessandro Busetti^1,†^, Ewa Wielogorska*^3^*, Olivier P. Chevallier*^3^*, Christopher T. Elliott*^3^,* Garry Laverty^1^, Sean P. Gorman^1^, William G. Graham^2^ and Brendan F. Gilmore^1, *^.

Table S1. Function, antibiotic resistance and cognitive HSL of strains used in study.

| **Strain** | **Genotype or phenotype** | **Cognitive HSL** | **Source/ references** |
| --- | --- | --- | --- |
| *A. tumefaciens* ATCC BAA 2240 | HSL sensor strain; contains traR and traG-lacZ fusion, a β-galactosidase reporter, β -lactam and Cn^R^ | C4-C12 3-oxo-HSLs 3-hydroxy- and 3-unsubstituted HSLs | 32 |
| *C. violaceum* CV026 | HSL sensor strain; miniTn-5 mutant of CV31532, violacein reporter, Km^R^ | C4-C8 HSLs. | 29 |
| *Escherichia coli* JM109 (pSB401) | HSL sensor strain; contains *luxRI::luxCDABE*, bioluminescent reporter, Tc^R^ | C6-C8-HSL and related medium side chain HSL analogues | 33 |
| E. coli JM109 (pSB 536) | HSL Sensor strain; contains *ahyR ahyI::luxCDABE*; Amp^R^ ColE1 origin | C4-HSLs and related short side chain HSL  analogues | 33 |
| *E. coli* JM109 (pSB 1142) | HSL sensor strain; contains *lasR*  and promoter of  *lasI::luxCDABE*, bioluminescent reporter, Tc^R^ | C10-C14 long side chain HSL analogues | 33 |
| *PAO-MW1* | *lasI* and *rhlI* mutant of *P. aeruginosa*, HgCl^R^ & Tc^R^ | BHL and OdDHL | 24 |

Table S2. UHPLC - MS/MS conditions for the analysis. Where: MW - Molecular weight, tr – Retention time.

| **Analyte** | **MW  [g/mol]** | **Moleculat Ion [M+H]^+^** | **Fragment Ions** | **tr** |
| --- | --- | --- | --- | --- |
| BHL | 171.09 | 172.1 | 102.1 | 1.83 |
|  |  |  | 71.1 |  |
|  |  |  | 154.0 |  |
| HHL | 199.12 | 200.0 | 102.2 | 3.67 |
|  |  |  | 74.1 |  |
|  |  |  | 99.2 |  |
|  |  |  | 71.2 |  |
| OHL | 227.15 | 228.0 | 102.2 | 5.46 |
|  |  |  | 74.1 |  |
|  |  |  | 109.1 |  |
| OdDHL | 297.19 | 298.0 | 102.2 | 6.91 |
|  |  |  | 98.2 |  |
|  |  |  | 95.1 |  |

Figure S1. Normalized bioluminescence of *E. coli* AHL bioluminescence reporters incubated with plasma exposed AHLs: BHL, HHL, OHL, OdDHL grown over 14 hours. AHL were exposed to a helium/oxygen 0.5% plasma plume for 0, 30 seconds, 60 seconds, 120 seconds, 240 seconds. (a) *E. coli* pSB 536 incubated with BHL at 30 ^o^C, (b) *E. coli* pSB 401 incubated with HHL at 37 ^o^C, (c) *E. coli* pSB 401 incubated with OHL at 37 ^o^C and (d) *E. coli* pSB 1142 incubated with OdDHL at 37 ^o^C. Each data point represents the mean of five replicates with error bars representing the standard deviation.

Figure S2. Optical Emission Spectra of the plume of a helium/oxygen (0.5%) plasma, operating at 6kV and 20 kHz.

Figure S3. Production of 2-hydroxyterephthalic acid (2-HTA) after plasma exposure of terephthalic acid in water and PBS. Each data point represents the average of three replicates with error bars depicting the standard deviation.

Figure S4. Production of hydrogen peroxide in PBS and water following plasma exposure using the titanium sulphate assay. Each data point represents the average of three replicates with error bars depicting the standard deviation.

Figure S5. Measurement of pH following plasma exposure of water (red squares) and PBS (black circles). A Hamilton mini-electrode pH meter was used for pH measurements. Each data point represents the average of three replicates with error bars depicting the standard deviation.

Figure S6. Plasma dependent temperature increase of PBS exposed to plasma over a series of time points. Each data point represents the average of three replicates with error bars depicting the standard deviation.


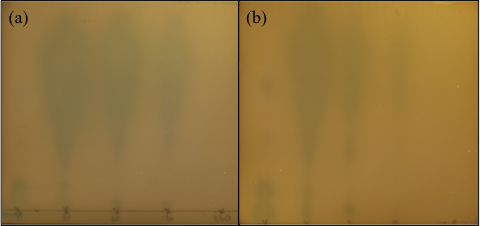


Figure S7. *A. tumefaciens* ATCC BAA-2240 TLC-overlay showing the effect of plasma exposure on AHL mobility and activity of OdDHL when exposed at a higher concentration of 1mM and in either (a) PBS or (b) water


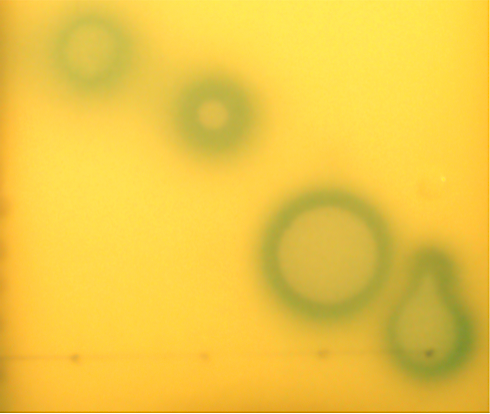

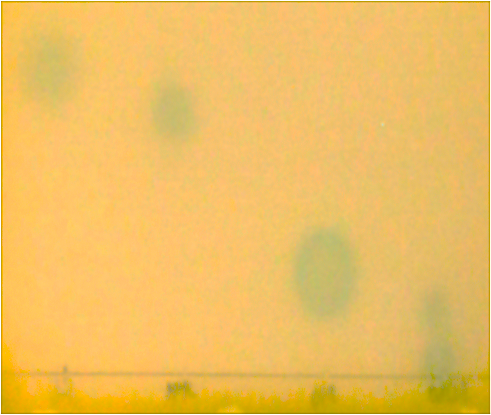


BHL

HHL

OHL

OdDHL

OdDHL

OHL

HHL

BHL

(a)

(b)

Figure S8. *A. tumefaciens* ATCC BAA-2240 TLC-overlay of AHLs (Grayscale) used in study (a) Untreated and (b) following incubation with 4 mM hydrogen peroxide for 240 seconds, showing negligible alteration in migration, compared with control.


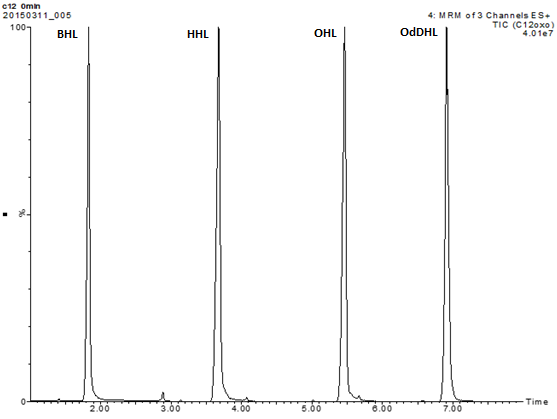


Figure S9 Overlay of LC - MS/MS chromatograms for 4 analytes at concentration of 1mM.

A.


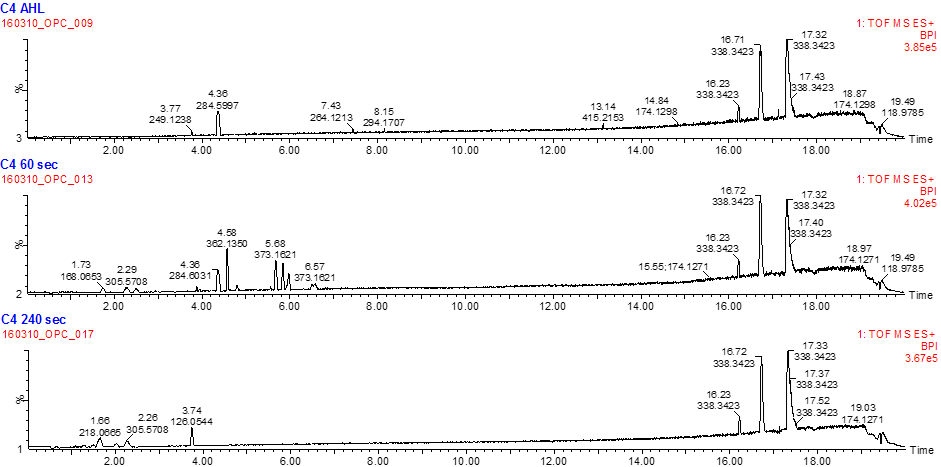


B.


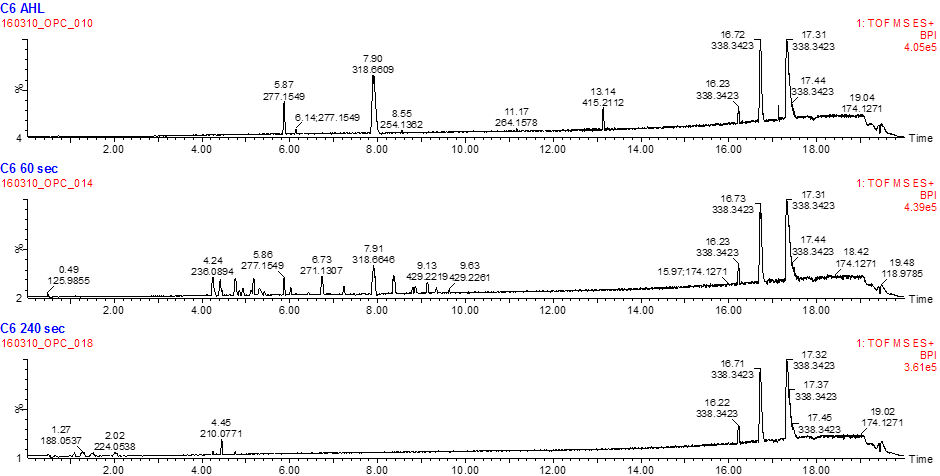


C.
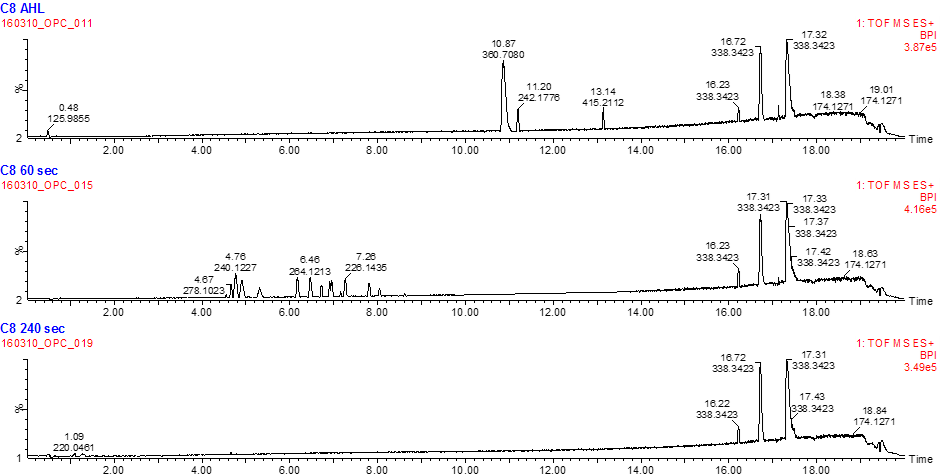


D.
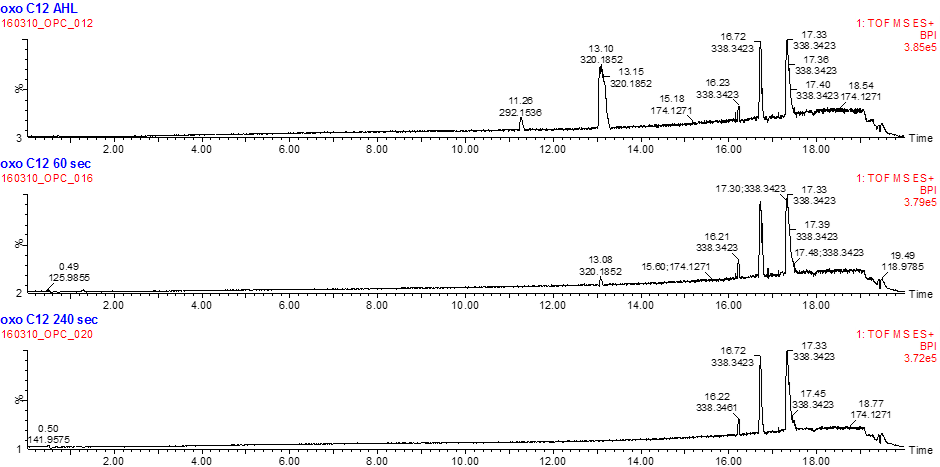


Figure S10 – LC-MS traces of BHL (A), HHL (B), OHL (C) and OdDHL (D) exposed to APNTP for (top to bottom) 0 seconds, 60 seconds and 240 seconds.

**Supplementary Materials and Methods**

**Optical Emission Spectroscopy And Chemical Characterization Of Plasma Treated Liquids.**

For optical emission spectroscopy the light of the plasma plume was focused onto a fiber optic using a lens (UV/SR-VIS, 300 μm in diameter). The fibre optic was connected to a broadband (200-1100 nm) spectrometer (Ocean optics, HR 4000, 0.25nm resolution) to disperse the collected light. The dispersed light and subsequent wavelengths were recorded using PC software (SpectraSuite®, Ocean optics) and reactive species produced within the plasma identified.

Titanium sulphate was used to colometrically determine the concentration of hydrogen peroxide produced in plasma treated water and PBS. 50 μl of deionised water or PBS was exposed to the plasma effluent for up to 240 seconds with measurements taken at intervals in triplicate. After exposure 10 μl was added to 90 μl of water in wells of a Nunc-96 well plate (Fisher Scientific UK Ltd. Loughborough, UK) followed immediately by the addition of 10 μl of a 60 mM sodium azide solution after which 50 μl of titanium sulphate was added. Absorbance of the yellow perxotitanium complex was read at 400 nm using a biotek microplate reader. (BioTek EL808; BioTek Instruments Ltd. Potton, UK) with H_2_O_2_ concentrations calculated from a standard curve.

Terephthalic acid (Sigma Aldrich, Dorset, UK) was used to evaluate the production of OH radicals in liquid. A 10 mM terephthalic acid, insoluble at acidic/neutral pH was created using a 5mM NaOH/PBS solution (pH 8) or 5mM NaOH/DI water which was then adjusted to pH 7.4 . 50 µl of this solution was exposed to the plasma effluent, 15 mm from the quartz tube tip to the surface of the sample for up to 240 second with measurements taken at intervals in triplicate. Unexposed samples were used as negative controls and correspond to time 0 seconds exposure. After exposure 20 µl of the exposed solution was transferred to 180 µl of PBS in a black bottomed 96-well plate (Fisher Scientific UK Ltd. Loughborough, UK). 2-Hydroxyterephthalic acid fluorescence was measured at emission 310 nm, excitation 425 nm using a BMG Fluostar Optima Fluorescence plate reader (BMG,Labtech Ltd. Aylesbury, UK). Fluorescence of 2-Hydroxyterephthalic from exposed samples were converted to concentrations using a standard curve of known concentrations of 2-Hydroxyterephthalic acid (Sigma Aldrich, Dorset, UK).

pH measurements of plasma exposed water and PBS were carried out immediately after transferring 20 µl of plasma exposed PBS or DI water to 180 µl of DI water. pH was measured using a Hamilton minitrode combination electrode pH meter, calibrated using pH 7 and pH 4 buffer solutions (Fisher Scientific UK Ltd., Loughborough, UK). Measurements were repeated in triplicate for each time point.

Temperature measurements of plasma treated liquids was investigated through the immersion of a Fluke CNX t3000 K-type temperature indicator (Fluke UK Ltd, Norwich, UK) in 50 µl of PBS and exposed for up to 240 seconds in triplicate. Temperature readings were taken at intervals for up to 240 seconds.

**AHL exposure to Hydrogen peroxide**

A concentration of 100 µM of each AHL was exposed to 4 mM of Hydrogen peroxide for 240 seconds at room temperature (equivalent to the concentration of hydrogen peroxide produced after four minutes plasma exposure). Briefly 1 mM AHL solutions were created as previously mentioned and added to 10 µl added to 90 µl of a 4 mM hydrogen peroxide solution in PBS. After 240 seconds hydrogen peroxide exposure 1 µl of each AHL was spotted onto RP-TLC paper and overlaid with *A. tumefaciens* ATCC-BAA 2240 as mentioned previously.

**LC-MS analysis**

Analyses were carried out on a Waters Acquity UPLC I-Class system (Milford, MA, USA) coupled to a Waters Xevo G2-S QTof mass spectrometer (Manchester, UK) with an electrospray ionisation source operating in positive with lock-spray interface for real time accurate mass correction. Instrument settings were as follow: source temperature was set at 120ºC, cone gas flow at 50 L.h^-1^, desolvation temperature at 450ºC, and desolvation gas flow at 850 L.h^-1^. The capillary voltage was set at 1.0 kV in positive mode. Source offset was 60 (arbitrary unit). Mass spectra data were acquired in continuum mode using MS^E^ function (low energy: 4 eV; high energy: ramp from 15 to 30 eV) over the range m/z 50-1200 with a scan time of 0.08 s. A lock-mass solution of Leucine Enkephalin (1 ng µL^-1^) in methanol/water containing 0.1% formic acid (1:1, v/v) was continuously infused into the MS via the lock-spray at a flow rate of 10 µL min^-1^.

The chromatographic separation was conducted on an Acquity HSS T3 column (100 mm x 2.1 mm, 1.8 µm). The column oven temperature was set at 45^o^C, injection volume at 5 µL and flow rate at 0.4 mL min^-1^. Mobile phase consisted of (A) water with 0.1% formic acid and (B) methanol with 0.1% formic acid. The gradient was set as follows: 1.50 min of 99% (A) followed by a linear increase from 1 to 99% (B) over 15 min, isocratic cleaning step at 99% (B) for 2 min, then returned to initial conditions 99% (A) over 0.25 min and column equilibration step at 99% (A) for 1.25 min.

Samples were reconstituted in H_2_O:MeOH (9:1, v/v) and 10 μL injected onto the system.
